# Analysis of social media compliance with cannabis advertising regulations: evidence from recreational dispensaries in Illinois 1-year post-legalization

**DOI:** 10.1186/s42238-023-00208-6

**Published:** 2024-01-03

**Authors:** Samantha Marinello, Rebecca Valek, Lisa M. Powell

**Affiliations:** 1 Division of Health Policy and Administration, School of Public Health, University of Illinois Chicago, 1603 W. Taylor Street, M/C 923, Chicago, IL 60612-4394 USA; 2 Oregon Health & Science University-Portland State University School of Public Health, 1810 SW 5th Ave, Portland, OR 97201-5200 USA

**Keywords:** Recreational cannabis, Social media, Advertising, Marketing

## Abstract

**Background:**

In the USA, an increasing number of states have legalized commercial recreational cannabis markets, allowing a private industry to sell cannabis to those 21 and older at retail locations known as dispensaries. Research on tobacco and alcohol suggests this new industry will use aggressive marketing tactics to attract new users and promote greater intensity of use. Of concern is that cannabis company advertising campaigns may be appealing to youth, promote false or misleading health claims, and disproportionately target low-income and minority communities. In this study, we evaluated recreational cannabis dispensary compliance with advertising regulations on social media in the state of Illinois.

**Methods:**

Primary data were collected from a census of recreational dispensary Facebook and Twitter business pages during the first year of recreational sales in 2020. A quantitative content analysis was conducted to systematically analyze the data; a codebook that detailed a protocol for classifying posts was developed prior to the analysis using advertising regulations outlined in the Illinois Cannabis Regulation and Tax Act. Violations of advertising regulations were organized into three categories: advertisements that may be appealing to youth (< 21 years old), advertisements that make health claims, and other advertising violations. The data were analyzed cross-sectionally and longitudinally. Additionally, differences in compliance were assessed by dispensary and neighborhood characteristics.

**Results:**

The results of the analysis revealed substantial and persistent non-compliance throughout the entire study period. Overall, nearly one third of posts had at least one violation and approximately one in ten posts met the criteria for appealing to youth or contained health claims. The majority of posts with health claims included health claims that were not qualifying conditions for medical cannabis access in the state of Illinois. No differences in compliance by neighborhood and dispensary characteristics were found.

**Conclusions:**

The findings from this study suggest that systematic monitoring and enforcement is needed to ensure compliance with advertising regulations.

## Background

In the USA, an increasing number of states have legalized commercial recreational cannabis markets, despite the fact that it is illegal to use or supply cannabis at the federal level. In these markets, private companies produce, distribute, and sell cannabis products to adults aged 21 and older at retail locations known as dispensaries. Discussions around public health regulations of recreational markets have drawn parallels to alcohol and tobacco and raise concerns about the effects of the developing industry’s marketing practices (Barry and Glantz 2017). A potential consequence of creating a legitimate, licit market for cannabis is that profit-driven companies will engage in promotional activities to attract new users and encourage greater intensity of use. Promoting heavy (daily or near daily use) and problem use (meeting the criteria for cannabis use disorder (Patel 2021)) is incentivized, as it is estimated that 80% of cannabis is currently consumed by these types of users (Caulkins et al. 2016). One potential consequence of legalization is greater underage youth exposure to marketing and advertising (Barry and Glantz 2017). Attracting youth is particularly desirable for companies because these customers are likely to yield lifelong dividends (Barry and Glantz 2017; Caulkins et al. 2016). Additionally, those who start using at a younger age are more likely to become heavy or problem users (Caulkins et al. 2016; Winters and Lee 2008). For example, it is estimated that half of all heavy users in the USA started using at age 14 or younger (Caulkins et al. 2016). Another concern is that the cannabis industry may follow in the footsteps of the tobacco industry and disproportionately target racial/ethnic minorities and low-income communities in their marketing campaigns (Truth Initiative 2020). For example, studies have found greater point-of-sale tobacco marketing (e.g., price discounts, branded advertisements), including marketing of menthol cigarettes, in Black and low-income communities (Cruz et al. 2019; Lee et al. 2015).

Decades of research on tobacco and alcohol have provided strong evidence that marketing exposure is causally related to initiation of use and regular use, particularly among youth (Anderson et al. 2009; DiFranza et al. 2006; Office of the Surgeon General 2014; Office of the Surgeon General 2012; National Cancer Institute 2008). Advertising by the tobacco industry has been shown to influence youth use through multiple channels, including by raising awareness of smoking, increasing brand recognition, reducing risk perceptions of use, and creating favorable beliefs surrounding use (Office of the Surgeon General 2012). A descriptive history of tobacco advertising reveals that there was intentional and persistent targeting of children and adolescents including, for example, promotions in schools, the use of cartoons, and endorsements from professional athletes (Pollay 1995).

Minimizing youth use of cannabis is an important public health objective (Pacula et al. 2014; Kees et al. 2020); adolescents are considered an at-risk population for cannabis use for multiple reasons. First, research indicates that early and frequent adolescent use negatively impacts brain development, leading to cognitive impairment in the domains of learning, attention, and memory (The National Academies and of Sciences, Engineering, and Medicine 2017). Second, adolescent use of cannabis is associated with poor social and educational outcomes including a decline in school performance, school dropout, unemployment, and use of other illicit drugs (Silins et al. 2014; Fergusson and Boden 2008). Lastly, evidence from longitudinal and cross-sectional studies have found cannabis use is associated with increased risk of mental illness, including depression (Silins et al. 2014; Gobbi et al. 2019), suicidal behavior (Silins et al. 2014; Gobbi et al. 2019), anxiety (Paruk and Burns 2016; Crippa et al. 2009), and psychosis (Paruk and Burns 2016), and, at the same time, youth with mental illness may use cannabis to self-medicate (Bottorff et al. 2009; Khantzian 1997).

Studies have shown that exposure to cannabis advertisements is already prevalent among adolescents. A large, nationally representative survey of 8th, 10th, and 12th graders found that 53% reported exposure to cannabis advertising online, 32% from television, 24% from magazines and newspapers, 20% from radio, 19% from storefronts, and 17% from billboards (Dai 2017). One study conducted in Oregon about 2 years after legal recreational sales began found exposure to advertising among youth was exceedingly common: 72% and 78% of 8th and 11th graders, respectively, reported seeing advertisements for cannabis, most commonly in storefronts and online (Fiala et al. 2020). Adolescent exposure to cannabis advertising is also associated with cannabis use (Dai 2017; Whitehill et al. 2020; D’Amico et al. 2018; D’Amico et al. 2015). Two longitudinal studies of adolescents in Southern California found greater exposure to medical cannabis advertising was associated with increased likelihood of cannabis use and greater intention to use one and seven years later (D’Amico et al. 2018; D’Amico et al. 2015). The study that followed adolescents for seven years also found that students with greater exposure were more likely to experience negative consequences of cannabis use, including missing school and having difficulty concentrating.

With the development of a recreational commercial market, there are also considerations for health claims in cannabis advertising. While cannabis meets the definition of a drug under the Food and Drug Administration’s (FDA) Food Drug and Cosmetic Act, it is not an FDA-approved drug, and the FDA does not allow companies to make health claims for drugs that are not FDA-approved (Caulkins 2017). However, in practice, the FDA does not punish companies for making health claims about cannabis products with tetrahydrocannabinol (THC) because cannabis is listed as a Schedule I drug under the Controlled Substance Act (Caulkins 2017). Therefore, the responsibility of regulating health claims in advertising falls on the states that legalized recreational markets.

Cannabis is distinct from tobacco and alcohol, the only two legal recreational drugs, in that it has been shown to have medical benefits. For example, according to the National Academy of Sciences, there is conclusive evidence that cannabis is effective at treating chronic pain, chemotherapy-induced nausea and vomiting, and multiple sclerosis (MS) spasticity symptoms (The National Academies and of Sciences, Engineering, and Medicine 2017). Many states with comprehensive medical cannabis programs also allow medical access for many conditions that are supported by low-quality evidence. For example, several states list opioid use disorder as an indication for medical cannabis access based on an ecological study that found medical cannabis legalization was correlated with a reduction in opioid overdoses (Shover et al. 2020). There are two concerns related to health claims in advertising of cannabis. First, without regulations and enforcement, dispensaries may make false or misleading health claims. Second, cannabis companies may promote the medical benefits of cannabis to create a “health halo effect,” which leads to positive perceptions of recreational use (Kees et al. 2020). For example, one major multi-state cannabis brand, MedMen, regularly touts the medical benefits of cannabis despite the fact that most of its stores sell only recreational cannabis (Ayers et al. 2019).

At the federal level, it is illegal to advertise cannabis because Section 843 of the Controlled Substance Act prohibits advertising of Schedule I drugs. Cannabis advertising regulations and enforcement, however, are set by states with legal markets because the Department of Justice allows state-sanctioned cannabis-related activities if states have “strong and effective regulatory and enforcement systems” that are consistent with federal enforcement priorities (Caulkins et al. 2015). All states with commercial recreational markets have regulations related to advertising and promotional activities, including restrictions on child-appealing marketing (Kees et al. 2020; Cao et al. 2020). However, the degree of systematic monitoring and enforcement of these regulations, as well as cannabis industry compliance, is unclear.

Currently, there is limited research on marketing practices of recreational dispensaries, including assessments of adherence to state regulations. Studies on this topic have been conducted in a variety of settings. A study examining point-of-sale marketing in recreational dispensaries in California found 35% had child-appealing marketing items in the interior of the store, 39% had signs, posters, or advertisements that promoted health benefits, and 22% violated regulations by providing free samples of cannabis products for customers to take away (Shi and Pacula 2021). Another study using the same audit tool in a subset of dispensaries located near schools found 74% had child-appealing marketing items in the interior of the dispensary. Furthermore, a qualitative content analysis of cannabis advertisements from freely distributed tabloids and magazines in Western Washington concluded that most advertisements contained themes that could be appealing to youth and focused on purported personal and social rewards from use (Carlini et al. 2020).

Electronic media, which is cited as the number one source of cannabis advertisements by adults and adolescents (Dai 2017; Krauss et al. 2017; Rup et al. 2020), is a third medium for advertising that has been evaluated. Studies have examined cannabis company advertising on their websites (Cavazos-Rehg et al. 2019; Bierut et al. 2017), on Weedmaps (Bierut et al. 2017) (a website that markets cannabis retailers online), and on social media platforms (Moreno et al. 2018; Jenkins et al. 2021; Spillane et al. 2021; Sheikhan et al. 2021). One study of medical and recreational dispensary websites in ten states found 75% did not have a method to verify the user was of legal age before they entered the site (Cavazos-Rehg et al. 2019). The results also revealed that most dispensary websites promoted health benefits of cannabis use (67%) (Cavazos-Rehg et al. 2019). A similar study of dispensary websites and Weedmaps webpages found many were not in compliance with state regulations in Colorado and Washington (Bierut et al. 2017). A large portion of websites did not have the required age gate (41% in Colorado and 35% in Washington) and 44% of Washington dispensaries made health claims, which is prohibited (Bierut et al. 2017). Two studies have examined content on social media from cannabis companies and found marketing strategies included references to popular culture to normalize use (Jenkins et al. 2021) and posts from cannabis vaporizer companies frequently showed someone using a product (68%) and had some posts with cartoons (5%) (Spillane et al. 2021). Two studies have examined cannabis company compliance with advertising regulations on social media platforms. The first study, which evaluated adherence to advertising regulations on Facebook, Twitter, and Instagram across all Canadian cannabis firms, reported that 86% of firms had at least one violation, most commonly involving lack of age restrictions, absence of information on health risks, and brand glamorization (Sheikhan et al. 2021). Additionally, 37.8% of firms made unsubstantiated health claims (Sheikhan et al. 2021). The second study used a sample of Facebook and Twitter posts from 38 recreational dispensaries in the state of Washington to examine compliance with state advertising regulations (Moreno et al. 2018). The study results revealed that very few posts were appealing to youth (0.01%), 13% of posts made health claims, which is prohibited, and 89% of posts lacked the required warning message (Moreno et al. 2018).

Social media is an important forum for dispensaries to market to current and potential consumers. While social media platforms such as Facebook and Twitter prohibit direct advertising of illegal drugs, cannabis companies can create promotional content on their social media business pages. Individuals can engage with these business pages in several ways: they can like, share, and comment on content as well as become a “follower” of the page. Furthermore, content promoted by the dispensaries on social media can be amplified by influencers and hashtags. Marketing on social media can potentially increase adolescent exposure to cannabis advertising because nearly all adolescents use social media platforms (Anderson and Jiang 2018). Recent lessons from the e-cigarette industry have demonstrated that social media campaigns with youth-oriented advertising can lead to a surge in use among youth (Jackler et al. 2019). Preliminary evidence also suggests that exposure to cannabis advertising on social media is associated with cannabis use (Whitehill et al. 2020; Trangenstein et al. 2019). The results of a study of adolescents and adults in Canada and the USA revealed that social media was the number one source of advertising and that advertising awareness and brand recall was associated with greater frequency of use (Rup et al. 2020). Another study of adolescents living in states with commercial markets found that adolescents who “liked” or “followed” a cannabis business on one or more social media platforms had five times greater odds of cannabis use in the past year (Trangenstein et al. 2019). Social media also has the potential to broadly disseminate unsubstantiated health claims. Because cannabis companies are not regulated by the FDA, they can make claims that are not supported by rigorous research (as required by the FDA); in fact, cannabis companies are known for publicizing low-quality, small-scale medical studies on social media (Caputi 2020).

The purpose of this study is to evaluate recreational dispensary compliance on social media with advertising regulations in the state of Illinois. On May 31, 2019, the Illinois General Assembly passed the Illinois Cannabis Regulation and Tax Act, which legalized the use and sale of cannabis starting January 1, 2020 (Cannabis Regulation and Tax Act 2019). Thirty-seven dispensaries began selling cannabis for recreational use on January 1; by the end of the year, the number of recreational dispensaries increased to 75. The Illinois Cannabis Regulation and Tax Act includes several restrictions on advertising, which it defines as engaging in any promotional activities, including through internet and electronic media. Cannabis companies are prohibited from using advertisements that (1) contain content that is likely to appeal to those under the age of 21 or (2) “make any health, medical, or therapeutic claims” (Cannabis Regulartion and Tax Act 2019). The Act also prohibits a number of other types of advertising practices, for example, promoting overconsumption and using images of cannabis leaf or bud (Cannabis Regulation and Tax Act 2019). Oversight and enforcement of these regulations are not designated to a specific regulatory body in the legislation; additionally, the legislation does not outline consequences for dispensary non-compliance (Cannabis Regulation and Tax Act 2019). To our knowledge, dispensary advertising practices are not systematically monitored for compliance with advertising regulations in the state of Illinois. In this study, primary data were collected from all recreational dispensary business pages on Facebook and Twitter for the entire year of 2020, the first year of recreational sales, to measure compliance with the Act. Understanding the current state of compliance is critical for informing future public health policies that aim to prevent advertising practices that may increase use among youth and misinform consumers about the health benefits and harms of cannabis.

## Methods

Data were collected during the first two weeks of each quarter of 2020 on all Facebook posts and Twitter tweets and retweets from the social media accounts of every recreational dispensary in Illinois (hereafter, posts, tweets, and retweets will be referred to as posts). To be included in the sample, an account must have represented at least one dispensary location in Illinois that sold recreational cannabis. Posts were only included in the analysis if they were posted while the dispensary was selling recreational cannabis. Accounts with fewer than 10 posts during the entire year were excluded from the analysis sample (*n* = 4). Some accounts represented multiple dispensary locations in Illinois (*n* = 16); in a few cases, accounts represented dispensary chains with locations in Illinois and in other US states (*n* = 5).

At each data collection time point, recreational dispensaries were identified using the Illinois Department of Financial and Professional Regulation’s Adult Use Cannabis website, which provides a list of all adult use dispensary licenses. Facebook and Twitter accounts of dispensaries were then identified using a search strategy that is detailed in Appendix A. Once the accounts were found, the dates of first legal recreational sales were identified using the protocol described in Appendix B. A description of the full analytic sample can be found in Table 1. Overall, 75 dispensaries sold recreational cannabis in 2020. Data were collected from 56 Facebook accounts, which represented 74 (98.6%) dispensary locations, and 11 Twitter accounts, which represented 32 (43%) dispensary locations. Among the sample with either a Facebook or Twitter account (*n* = 74), all of them had a Facebook account, and thus, overall, 43% of dispensaries were represented by both a Facebook and Twitter account. A total of 10,461 posts (7793 from Facebook and 2668 from Twitter) were included in the full analytic sample.

**Table 1:** Summary of full analytic sample

	*N* (%)
Recreational dispensaries	75 (100%)
Facebook accounts	56
Dispensary representation	74 (98.6%)
Twitter accounts	11
Dispensary representation	32 (42.7%)
Observations	10,461 (100%)
Facebook posts	7,793 (74.4%)
Tweets	2,463 (23.5%)
Retweets	205 (2.0%)

Data were collected for the entire year of 2020

Portable Document Format (PDF) files of dispensary Facebook and Twitter pages were generated using two software programs, NCapture and Fireshot Pro. NCapture, which is an extension of NVivo, the qualitative data analysis software used to analyze the data, was used to collect Facebook data in the first half of 2020. In the second half of 2020, NCapture could not be used because Facebook made changes to its Application Programming Interface in September of 2020. Fireshot Pro, a web page screenshot software, was used to collect the remaining data on Facebook as well as all Twitter page data. For each dispensary social media page, a single PDF that included all posts was compiled. All PDFs included live links (e.g., links to articles or videos).

A quantitative content analysis was used to systematically analyze the data. Each post was reviewed and coded independently by two coders based on its compliance with advertising regulations outlined in the Illinois Cannabis Regulation and Tax Act (Cannabis Regulation and Tax Act 2019). A codebook (see Appendix C, Table 5A) that detailed a protocol for classifying posts was developed a priori using language from the law. All discrepancies in coding were reconciled through consensus after both coders re-reviewed the data. Articles and videos attached to posts were read/watched if they were related to health claims, product reviews, consumption, or different cannabinoids (e.g., THC). Posts were uniquely identified by the date they were published online (unique identifiers were further generated if multiple posts occurred in a day) and could be coded under multiple codebook categories. All data were analyzed in NVivo software.

Violations of advertising regulations were assessed based on three types of violations: advertisements that may be appealing to youth (< 21 years old), advertisements that make health claims, and other advertising violations.

For advertisements that may be appealing to youth, seven categories were used in the primary analysis based on the legislation: (1) images of or statements about animals, (2) cartoons, (3) images of toys, (4) images of or statements about children, (5) product imitation of candy packaging or labeling, (6) promotional activities that may appeal to children, and (7) any other likeness to images, characters, or phrases that may be appealing to children (Cannabis Regulation and Tax Act 2019). In addition to the categories based on the law, four other categories were developed: (1) images of or statements about famous people (e.g., professional athletes, actors, and musicians), (2) name of strain that could be appealing to youth, (3) professional images of young adults, and (4) dispensary-sponsored activities that could be appealing to adolescents.

Health claims could be related to any cannabis chemical compound found in consumable products, including THC, cannabidiol (CBD), terpenes, and other cannabinoids. Additionally, indirect health claims about cannabis policies were included (e.g., states that legalized cannabis saw a decrease in opioid overdose deaths). Symptoms related to specific diseases were only coded under that disease (e.g., reduce pain for people with MS was coded under MS but not pain). In total, the codebook included 72 unique health claims, many of which were added to the codebook after analyzing the data in the first quarter of 2020. Health claims were further classified as qualifying and non-qualifying conditions for medical cannabis access in Illinois.

Other violations of advertising regulations based on the law that were included in this analysis were (1) the depiction of consumption, (2) promotion of overconsumption, (3) inclusion of an image of cannabis leaf or bud, (4) giving away cannabis products for free, (5) games or competitions related to consumption, and (6) the bundling of multiple products for one price (Cannabis Regulation and Tax Act 2019).

Differences in compliance by neighborhood and dispensary characteristics were assessed. This assessment was undertaken only for dispensaries that had Facebook posts (*n* = 74) given that all dispensaries in the sample were represented by a Facebook account. The characteristics included were median household income, percentage of the population that is Black or Hispanic, and whether a dispensary sold medical and recreational cannabis or just recreational cannabis (hereafter referred to as dispensary type). Census-tract level 5-year estimates on median household income and the percentage of the population that is Black or Hispanic were collected from the American Community Survey for 2015–2019. Dispensaries were classified into three race/ethnicity and income categories based on tertiles. For race/ethnicity, the tertile groups were < 9.9%, ≥ 9.9% and < 24.4%, and ≥ 24.4% Black or Hispanic. The income tertile groups were household median income < $51,298, ≥ $51,298 and < $77,528, and ≥ $77,528.

Descriptive statistics were used to summarize compliance with regulations in the first year of legal recreational sales. The data were analyzed cross-sectionally and longitudinally. For the cross-sectional analysis, the number and percentage of posts with violations were calculated for each compliance category. Additionally, the number and percentage of social media accounts and dispensaries with at least one violation were computed. Differences in non-compliance rates between posts on Facebook and Twitter were evaluated using two proportion *z*-tests. For the longitudinal analysis, non-compliance rates were calculated for each month of the year. We examined changes in non-compliance over time given that (1) non-compliance may have increased over time as the market developed and expanded since more recreational dispensaries opened and dispensaries would have more time to employ more sophisticated marketing strategies or (2) non-compliance may have decreased over time as dispensaries became more aware of Illinois advertising regulations.

Non-compliance by neighborhood and dispensary characteristics were evaluated using Facebook account data. Non-compliance rates were first calculated for each Facebook account. Then, account-level non-compliance rates were added to the dispensaries represented by each account. Finally, non-compliance rates by area race/ethnicity, income, and dispensary type categories were computed using the dispensary-level data. Adjusted and unadjusted linear regression models were used to assess whether non-compliance differed by income tertiles, race/ethnicity tertiles, and dispensary type. Post-regression *t*-tests were also used to examine pairwise comparisons across tertile groups, and *p*-values for these tests were adjusted for multiple comparisons using the Bonferroni method. Non-compliance outcomes included percentage of posts with (1) any violation, (2) content that may be appealing to youth, (3) a health claim, and (4) an “other” violation.

## Results

Table 2 provides results for the cross-sectional analysis, and Table 6A, Appendix D, compares compliance on Facebook and Twitter. Overall, 30.6% of posts included at least one violation; 9.3% of posts were coded as appealing to youth, 10.7% of posts included health claims, and 16.7% were coded as having an “other” violation. When including the additional appealing to youth categories that were not listed in the legislation, the percentage of posts that were coded as appealing to youth increased to 13.9%. By social media account, there was significant variation in the proportion of posts with violations (range: 0.0–50.3%) as well as the number of posts with violations. For example, the top ten social media accounts with the most violations (15% of all accounts) were responsible for 51% of violations.

**Table 2:** Cross-sectional analysis: violations of advertising and sales regulations for posts, accounts and dispensaries, by violation type

	Posts, *n* (%)	Accounts, *n* (%)	Dispensaries, *n* (%)
Total sample size	*N* = 10,461	*N* = 67	*N* = 75
Any violation	3,202 (30.6%)	66 (98.5%)	74 (98.6%)
Appealing to youth	976 (9.3%)	63 (94%)	70 (93.3%)
Animal	488 (4.7%)	57 (85.1%)	63 (84.0%)
Cartoon	432 (4.1%)	54 (80.1%)	67 (89.3%)
Child	61 (0.6%)	18 (26.9%)	23 (30.2%)
Toy	43 (0.4%)	25 (37.3%)	38 (50.7%)
Likeness	192 (1.8%)	37 (55.2%)	35 (46.7%)
Packaging	0 (0.0%)	0 (0.0%)	0 (0.0%)
Activity appealing to child	8 (0.1%)	5 (7.5%)	7 (9.3%)
Health claim	1,115 (10.7%)	59 (88.1%)	67 (89.3%)
Non-qualifying conditions	651 (6.2%)	58 (86.6%)	67 (89.3%)
Top 10 health claims			
1. Anxiety	224 (2.4%)	38 (56.7%)	46 (61.3%)
2. Pain	216 (2.1%)	33 (49.2%)	43 (57.3%)
3. Relaxing	183 (1.8%)	38 (56.7%)	53 (70.7%)
4. Sleep	165 (1.6%)	40 (59.7%)	51 (68.0%)
5. Chronic pain	140 (1.3%)	29 (43.3%)	43 (57.3%)
6. Improve mood	131 (1.3%)	36 (53.7%)	43 (57.3%)
7. Inflammation	122 (1.2%)	27 (40.3%)	34 (45.3%)
8. Stress	108 (1.0%)	26 (38.9%)	37 (49.3%)
9. Cancer	95 (0.9%)	19 (28.4)	25 (33.3%)
10. Depression	92 (0.9%)	18 (26.9%)	28 (37.3%)
Other violations	1743 (16.7%)	65 (97.0%)	73 (97.3%)
Depicts consumption	197 (1.9%)	32 (47.8%)	33 (44.0%)
Image of leaf or bud	1,532 (14.6%)	64 (95.5%)	73 (97.3%)
Promotes overconsumption	24 (0.2%)	9 (13.4%)	11 (14.7%)
Bundling products for one price	14 (0.1%)	11 (16.4%)	14 (18.7%)
Giving away products	53 (0.5%)	11 (16.4%)	11 (14.7%)
Conducting games/competitions	0 (0.0%)	0 (0.0%)	0 (0.0%)

Data were collected for the entire year of 2020. Rows do not sum to total violation in a category because each post could be coded into multiple categories. Number of accounts includes all accounts with at least one violation. Number of dispensaries includes all dispensaries represented by the Facebook and/or Twitter accounts with at least one violation. In some cases, social media accounts can represent multiple dispensary chain locations. Dispensaries with both Facebook and Twitter accounts can have a total of two accounts

The most common reason posts were coded as appealing to youth were that they included statements about or images of animals (4.7% of all posts, representing 50% of youth appealing posts) and/or included a cartoon (4.1% of all posts, representing 42% of youth appealing posts). The top ten health claims were related to anxiety, pain, relaxation, sleep, chronic pain, improved mood, inflammation, stress, cancer, and depression. Overall, 58% of posts with health claims included health claims that were not qualifying conditions for medical cannabis access in Illinois. Examples of unsubstantiated health claims were weight loss, diabetes prevention, increased energy, improved exercise, treatment for COVID-19, and treatment for hypertension. The most common reason advertisements were coded under other violation was that the post included an image of cannabis leaf or bud (14.6% of all posts, representing 88% of posts coded as “other” violation). The second most common reason was that the post included an image or video depicting consumption (2.1% of all posts, representing 11% of posts coded as “other” violation). There were very few or zero posts that were coded under the remaining “other” categories. Non-compliance rates on Facebook were consistently higher than those on Twitter across violation types; nearly all differences in non-compliance were statistically significant at the 5% significance level (see Appendix D, Table 6A).

**Fig. 1:**
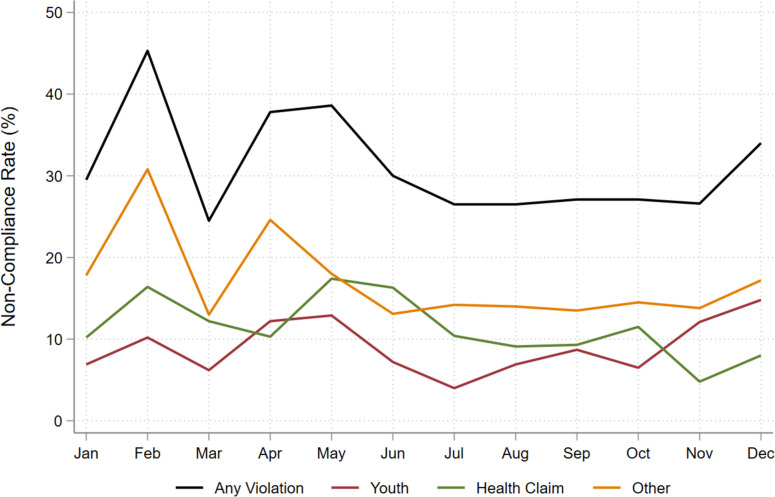
Monthly non-compliance rates by violation type in 2020 A total of *N* = 10,461 posts were assessed in the analysis

Monthly non-compliance summary statistics are provided in Table 3. Figure 1 shows monthly overall non-compliance and non-compliance rates by violation type. The results revealed that non-compliance remained relatively constant over time—in most months the percentage of posts with one or more violations was about 30% with a range of 24–45%. Overall non-compliance rates by neighborhood and dispensary characteristics are shown in Table 4. Regression analyses and paired *t*-tests did not reveal differences in non-compliance by area race/ethnicity or income tertiles or by dispensary type that were statistically significant at the 5% significance level.

**Table 3:** Longitudinal analysis: monthly changes in non-compliance rates in 2020

*n* = posts, accounts	*n* = 824, 42	*n* = 627, 47	*n* = 917, 48	*n* = 883, 46	*n* = 599, 50	*n* = 720, 54	*n* = 748, 53	*n* = 826, 55	*n* = 1,000, 59	*n* = 861, 60	*n* = 1,178, 63	*n* = 1,278, 65
	**January**	**February**	**March**	**April**	**May**	**June**	**July**	**August**	**September**	**October**	**November**	**December**
	(%)	(%)	(%)	(%)	(%)	(%)	(%)	(%)	(%)	(%)	(%)	(%)
**Any violation**	29.5%	45.3%	24.5%	37.8%	38.6%	30.0%	26.5%	26.5%	27.1%	27.1%	26.6%	34.0%
**Appealing to youth**	6.9%	10.2%	6.2%	12.2%	12.9%	7.2%	4.0%	6.9%	8.7%	6.5%	12.1%	14.8%
**Health claim**	10.2%	16.4%	12.2%	10.3%	17.4%	16.3%	10.4%	9.1%	9.3%	11.5%	4.8%	8.0%
**Other violations**	17.8%	30.8%	13.0%	24.6%	18.0%	13.1%	14.2%	14.0%	13.5%	14.5%	13.8%	17.2%

**Table 4:** Percentage of posts with violations by neighborhood and dispensary characteristics

	Any violation	Appealing to youth	Health claim	Other violations
Percentage Black or Hispanic				
Tertile 1 < 9.9%, *n* = 25	26.8%	9.3%	9.4%	13.2%
Tertile 2 ≥ 9.9% and < 24.4%, *n* = 25	29.7%	11.1%	6.6%	15.8%
Tertile 3 >24.4%, *n* = 24	27.1%	8.4%	6.8%	15.1%
Median household income				
Tertile 1 < $51,298, *n* = 24	27.1%	10.0%	6.1%	14.2%
Tertile 2 ≥ $51,298 and < $77,528, *n* = 25	29.8%	9.4%	9.7%	16.2%
Tertile 3 ≥ $77,528, *n* = 25	26.8%	9.5%	6.9%	13.6%
Dispensary type				
Medical and recreational, *n* = 46	27.0%	8.9%	7.4%	14.6%
Recreational only, *n* = 28	29.4%	10.8%	7.9%	14.8%

Data were collected for the entire year of 2020. Analysis was conducted at the dispensary level. Data from one social media account could be used for multiple dispensaries if that account represented multiple retail locations. Linear regression models were used to examine differences in non-compliance outcomes by dispensary neighborhood race/ethnicity and income as well as dispensary type. Additionally, adjusted regression models that included neighborhood income tertiles, race/ethnicity tertiles, and dispensary type were used. Pairwise *t*-tests were employed to compare means across tertile groups for models with income and race/ethnicity tertiles. *p*-values were adjusted for multiple comparisons using the Bonferroni method. None of the differences in means were statistically significant at the 5% significance level

## Discussion

This study evaluated recreational cannabis dispensary compliance with advertising regulations in Illinois on Facebook and Twitter. Overall, we found non-compliance was common and persisted over time: nearly one third of posts were not in compliance with the Illinois Cannabis Regulation and Tax Act throughout the entire 2020 study period. Approximately one in ten posts were in violation based on meeting the criteria for appealing to youth according to the examples provided in the legislation. This is likely an underestimate of content that is appealing to youth for two reasons. The first is that including the additional criteria for appealing to youth (celebrities, strain names that may be appealing to youth, professional images of young adults, and dispensary-sponsored activities that could be appealing to adolescents) increased the non-compliance rate to 13.9%. The second reason is that many edibles, which often look just like candies and other sweets (e.g., lollipops, gummy bears, chocolate bars), are likely appealing to youth.

Health claims, especially unsubstantiated health claims, were also a common violation: about 11% of posts included health claims and 58% of posts with health claims were not qualifying conditions for medical cannabis access in Illinois. The fact that social media accounts for recreational only dispensaries were just as likely as medical and recreational dispensary accounts to make health claims is revealing. This finding suggests recreational only dispensaries promote medical benefits (both legitimate and unsubstantiated) of cannabis as a marketing tool to encourage recreational use. In terms of other violations (prevalence of about 17%), images of cannabis leaf of bud made up the vast majority of these violations.

The results revealed no differences in non-compliance by dispensary and neighborhood characteristics. One explanation is that several social media accounts (31%) represent dispensary chains with multiple locations. In some cases, these chains were located in areas with diverse racial and income compositions. This finding suggests dispensaries/dispensary chains may not be disproportionately targeting low-income and minority communities with advertisements that violate regulations on their Facebook and Twitter pages.

The findings from this study are mostly consistent with the literature on marketing practices of recreational dispensaries, which have found that non-compliance with marketing regulations is common (Shi and Pacula 2021; Bierut et al. 2017; Moreno et al. 2018; Sheikhan et al. 2021). However, it is difficult to directly compare our findings for content that is appealing to youth to previous studies because there is variation in how child/youth-appealing marketing is defined. For example, some studies included bright colors, bubble-like fonts, and products that are typically consumed by children (e.g., candy) in their definition of content that is appealing to youth (Cao et al. 2020; Shi and Pacula 2021). Another issue is that sometimes definitions provided by studies were vague and therefore subjective (Moreno et al. 2018). However, our finding that dispensaries engage in marketing that is appealing to youth (9.3% of all posts) is consistent with results from two point-of-sale studies in California and a study of print media in Western Washington (Cao et al. 2020; Shi and Pacula 2021) but differs from a study conducted in Washington that found very few (0.01%) of social media posts were appealing to youth (Moreno et al. 2018). Results from the two point-of-sales studies suggest recreational dispensaries are more likely to have child-appealing marketing in the interior stores that are near schools (74% vs. 35% of dispensaries), and the print media study found that tabloids and magazine cannabis advertisements include themes that are appealing to youth (Cao et al. 2020; Shi and Pacula 2021; Carlini et al. 2020). In terms of health claims, this study finds dispensaries often make health claims (11% of posts; 89% of dispensaries), which is consistent with point-of-sale studies (39% of California dispensaries (Shi and Pacula 2021); 44% of California dispensaries near schools (Cao et al. 2020), studies of dispensary websites (67% of dispensaries (Cavazos-Rehg et al. 2019); 61% of dispensaries in Colorado and 44% in Washington (Bierut et al. 2017)), and studies of social media (13% of posts (Moreno et al. 2018); 37.8% of firms make unsubstantiated health claims (Sheikhan et al. 2021)).

The non-compliance results found in this study are concerning for multiple reasons. The first is that exposure to marketing, particularly marketing that is appealing to youth, may increase initiation of use and frequency of use among youth, who are very likely to have social media accounts (Anderson and Jiang 2018) and experience greater health harms from cannabis use (The National Academies and of Sciences, Engineering, and Medicine 2017; Silins et al. 2014; Fergusson and Boden 2008; Gobbi et al.^ 2019^). Research from tobacco, alcohol, and e-cigarettes have demonstrated that exposure to marketing is causally related to initiation of use and regular use (Anderson et al.^ 2009^; DiFranza et al. 2006; Office of the Surgeon General 2014; Office of the Surgeon General 2012; National Cancer Institute 2008; Jackler et al.^ 2019^), and preliminary evidence suggests exposure to cannabis advertising, including advertising on social media, is linked to use (Dai 2017; Whitehill et al. 2020; D’Amico et al. 2018; D’Amico et al. 2015). A second concern is that dispensaries are using social media to widely disseminate false or misleading health claims. This could be particularly dangerous if people forgo effective health treatments and instead use cannabis. One example is depression, which was one of the top health claims made by dispensaries. While there are many effective treatments for depression, multiple systematic reviews and meta-analyses have found that cannabis use is associated with the development of depressive disorders and suicidality (Lev-Ran et al. 2014; Moore et al. 2007; Borges et al. 2016). Consumers who are used to the rigorous standards set by the FDA for health claims in the pharmaceutical industry may be deceived by health claims made in the cannabis industry that are supported with little to no evidence (Caputi 2020). False or misleading health claims about cannabis also has the potential to influence use; for example, evidence from the tobacco literature suggests that fraudulent claims that filtered cigarettes were “healthier” than unfiltered cigarettes lowered the perceived risks of smoking, reduced smoking cessation, and increased cigarette sales (Pollay and Dewhirst 2002; Warner 2002; Silva et al. 2021). A third concern is that dispensaries appear to be using legitimate health benefits as a marketing tool to promote recreational use. This type of marketing may reduce risk perceptions of cannabis use by insinuating that cannabis use is harmless or even healthy. False and misleading health claims made by dispensaries may have a greater impact on cannabis use by youth, particularly mentally ill youth, because youth in general are more susceptible to marketing and advertising (Lapierre et al. 2017; Pechmann et al. 2005) and evidence shows that youth may use a cannabis as a form of self-medication to alleviate symptoms of mental illness (Bottorff et al. 2009; Khantzian 1997). A potential solution for this problem (in addition to enforcement of current regulations that prohibit health claims) is to require health warnings on all advertisements.

Because violations were common and persisted over time, it is likely that advertising regulations are not systematically enforced on social media in the state of Illinois. In order to reduce advertising violations, enforcement must be designated to a regulatory body that has the resources to monitor dispensary marketing activities, especially online because online advertisements are the number one source of advertisement exposure (Dai 2017; Krauss et al. 2017; Rup et al. 2020). Additionally, penalties may be needed to ensure compliance. For example, fines for violations could be imposed on dispensaries, which can also be used to fund ongoing monitoring. Another potential solution is to create a mechanism for which people can easily report violations to regulatory authorities.

A strength of this study is the use of the universe of Facebook and Twitter data for an entire year. The fact that all data were double coded by two independent researchers also ensures validity and reliability across dispensaries over time. To our knowledge, this is the first study to examine changes in compliance over time and differences in compliance by neighborhood and dispensary characteristics. Additionally, unlike previous studies of compliance on social media, this study codes specific health claims and reasons why posts are appealing to youth.

This study has several limitations. First, it only assessed activity on Facebook and Twitter, when other social media platforms like TikTok and Instagram may have larger followings by youth. However, Facebook and Twitter are widely used by adults and youth. In 2021, it was estimated that 69% and 23% of adults used Facebook and Twitter, respectively; in 2018 and 2022, 51% and 32% of adolescents used Facebook (Gramlich 2021; Vogels et al. 2022), respectively, and 32% and 23% used Twitter, respectively (Anderson and Jiang 2018; Vogels et al. 2022). Additionally, Facebook and Twitter use was higher among lower-income adolescents compared to higher income adolescents (44% vs. 27% on Facebook and 26% vs. 22% on Twitter in 2022) (Vogels et al. 2022). As new social media platforms emerge over time and change in their popularity, it will be important that future studies examine these sources. Second, because data were only collected for 1 year, it is impossible to separate the overall trend from seasonal trends. Third, it was difficult to evaluate differences across neighborhoods, as nearly one third of accounts represented more than one dispensary location. Lastly, we did not track the number of followers or characteristics of followers over time or evaluate engagement with social media posts, such as Facebook likes and comments and Twitter likes, retweets, and comments. Evaluating followers and tracking level of engagement are important for understanding dispensaries’ reach and influence.

## Conclusions

This study provided a comprehensive evaluation of recreational cannabis dispensary compliance with advertising regulations in the state of Illinois in the first year of recreational sales. Overall, we observed substantial (30.6%) non-compliance that persisted throughout the entire year. Of concern are advertisements that are appealing to youth and advertisements that make health claims, especially unsubstantiated health claims that may affect consumers’ healthcare decisions. This study is important for policymakers in Illinois as well as policymakers in states considering legalizing recreational cannabis or improving regulations surrounding cannabis advertising. Future work is needed to assess compliance over a longer period and on other social media platforms. Additionally, research that tracks the number of dispensary followers, engagement with followers, and characteristics of followers is needed to understand the influence of marketing efforts as well as potential differential impacts by follower demographics.

## Data Availability

The datasets used and/or analyzed during the current study are available from the corresponding author on reasonable request.

## Appendix A

Protocol for Identifying Social Media Accounts

1. Search for dispensary name on Facebook and Twitter. If the dispensary is a chain with multiple locations, always include the location
  a Ex. “Herbal Remedies dispensary”, “Sunnyside Lakeview”
2. Ensure it is their official social media by reviewing the “About” section on Facebook and the Twitter bio
  a Is the location correct?
  b Does it include their official logo (see website)?
  c Do they have a link to their official website?
3. Go to dispensary website to look for links to social media (usually small icon for FB and Twitter). If you did not find their social media accounts by searching on Facebook and Twitter, you can sometimes find the page through their website. If you did find their social media page by searching, check if the link on their website brings you to the same page.
  a Note that sometimes websites do not include links to social media pages or the links are outdated.
4. If you could not find their page by searching on FB/Twitter or through their website, search for their social media accounts through Google. Use the name of the dispensary and social media platform. Try the search with and without the location. If the dispensary is a chain with multiple locations, always include the location.
  a Ex. “MOCA Modern Cannabis Facebook”, “Dispensary 33 Twitter”
  b Ex. “MOCA Modern Cannabis Chicago Facebook”, “RISE Naperville Facebook”
5. If a location-specific account in Illinois does not exist for a dispensary chain, use their general Illinois or US social media account if it exists (repeat steps 1–4)
  a Ex. Beyond/Hello has two dispensaries in Illinois that share a single Facebook page.

## Appendix B

Protocol for identifying first day of legal recreational sales

1. Conduct a Google search that includes the name of the dispensary, dispensary location, and “first day of recreational marijuana sales.”
2. Identify 1–2 sources that report the exact date that recreational sales began. Sources can include news articles, dispensary web pages, and dispensary social media pages.
3. Archive sources using the Way Back Time Machine and save the archived links.

## Appendix C

**Table 5:** Codebook for quantitative content analysis

Variable	Description	Violation type
Animals	Images of animals; statements about animals Also includes: cartoons and drawings; illustrations of insects (like bees and caterpillars) if they look like they might be appealing to children Exclude: all other images of insects Examples: Photo of dog, cartoon character of any animal (e.g., dog, cat, Garfield, Kermit the Frog)	Appealing to youth
Likeness	Likeness to images, characters, or phrases that are designed in any manner to be appealing to or encourage consumption of persons under 21 years of age. Also, images, characters, or phrases that are popularly used to advertise to children Also includes: references to characters, TV shows, or movies that are watched by children Exclude: images of edibles like cookies, brownies, gummies unless they look like products that are specifically marketed to children (e.g., gummies that look like animals, gummies that look like Sour Patch Kid candies) Examples: Image of edible candy next to brands of candy that are sold to children (e.g., Starbursts, M&M’s, Sour Patch Kids) “May the terps always be in your favor” with an image of a Star Wars character	Appealing to youth
Name	Name of strain (flower, vaporizer, or concentrate) that could be appealing to children Exclude: edibles Examples: Applejacks, Girl Scout Cookies, Juicy Fruit, blueberry pie, bubblegum, cookies and cream, mango	Appealing to youth
Child	Image of child; statements about children or references to children (< 18 years old) Excludes: posts about children with illnesses that are treated with cannabis (e.g., autism, epilepsy) Examples: How to talk to your children about cannabis, image of a family that includes a child	Appealing to youth
Cartoons	Drawing or depiction of object, person, animal, creature, or similar caricature that has exaggerated features, applying human characteristics to animals or inanimate objects, or giving extra-human abilities/super powers. Also, any cartoon characters from a TV show that children watch. Excludes: standard emojis (e.g., yellow smiley face) Examples: Scooby Do, Snoopy, drawing of pot leaf with googly eyes, image of animal with a hat, drawing of person flying	Appealing to youth
Packaging	Any packaging or labeling that bears reasonable resemblance to any product available for consumption as a commercially available candy, or that promotes consumption of cannabis Example: Edible chocolates that look like Hersey’s kisses	Appealing to youth
Toy	Image of a toy Also includes: Photos, cartoons, and drawings Excludes: bicycle, sporting equipment (e.g., bat, soccer ball). The exception is a child’s bike or sporting equipment (t-ball) Examples: Doll, stuffed animal, G.I. Joe, action figure, LEGOS, hot wheels, Crayons, Play-Doh, board games usually played by children (Candy Land), children’s book	Appealing to youth
Famous	People who are famous, including musicians, actors, professional athletes, comedians, and reality stars Excludes: Politicians unless they meet the inclusion criteria (e.g., Arnold Schwarzenegger) Examples: Snoop Dogg, Dave Chappelle, Kim Kardashian, Michael Phelps	Appealing to youth
Young_adult	Professional images of young adults (~21–25) Excludes: Pictures with multiple people of all age groups and pictures of employees or customers	Appealing to youth
Activity_child	Promotional activities or events that could be appealing to children (National Cancer Institute 2008) Examples: Ice cream social, magic show, and carnival games	Appealing to youth
Activity_youth	Promotional activities or events that could be appealing to adolescents (Pollay 1995; Pacula et al. 2014; Kees et al. 2020; The National Academies and of Sciences, Engineering, and Medicine 2017; Silins et al. 2014; Fergusson and Boden 2008; Gobbi et al. 2019; Paruk and Burns 2016) Examples: Festival, concert	Appealing to youth
Anxiety	Makes any health, medical, or therapeutic claims related to anxiety. Excludes: Stress and worrying	Health claim
Stress	Makes any health, medical, or therapeutic claims related to stress Excludes: Anxiety and worrying Example: “Certain strains of cannabis are best for reducing stress”	Health claim
Depression	Makes any health, medical, or therapeutic claims related to depression Example: “Studies suggest CBD is a fast-acting anti-depressant”	Health claim
PTSD	Makes any health, medical, or therapeutic claims related to post-traumatic stress disorder (PTSD) Example: “One study suggests that cannabis can reduce PTSD symptoms by 50%”	Health claim
Epilepsy	Makes any health, medical, or therapeutic claims related to epilepsy Example: “Epilepsy is highly responsive to weed”	Health claim
Diabetes	Makes any health, medical, or therapeutic claims related to diabetes Example: “Using cannabis has been shown to prevent diabetes”	Health claim
Cancer	Makes any health, medical, or therapeutic claims related to treating the symptoms of cancer or symptoms from cancer treatment Also includes: statements about cannabis being “anti-cancer” Excludes: Claims only related to killing cancer cells	Health claim
Cells	Makes any health, medical, or therapeutic claims related to killing/preventing growth of tumor or cancer cells Excludes: Ambiguous comments of “anti-cancer” or “anti-tumor”	Health claim
Sleep	Makes any health, medical, or therapeutic claims related to sleep Excludes: General statements about sedation that does not include sleep	Health claim
Insomnia	Makes any health, medical, or therapeutic claims about insomnia	Health claim
Chronic_pain	Makes any health, medical, or therapeutic claims related to chronic pain Also includes: Statements about using cannabis for pain management Excludes: Pain that is a result of another illness (these will only be coded under that illness)	Health claim
Pain	Makes any health, medical, or therapeutic claims about general pain Excludes: Chronic pain or pain from an illness/disease/condition Example: “Cannabis can help with everyday aches and pains”	Health claim
MS	Makes any health, medical, or therapeutic claims related to multiple sclerosis	Health claim
Hypertension	Makes any health, medical, or therapeutic claims related to hypertension Example: “A new study finds CBD can lower your blood pressure”	Health claim
ADHD	Makes any health, medical, or therapeutic claims related to attention hyperactivity deficit disorder (ADHD)	Health claim
Autism	Makes any health, medical, or therapeutic claims related to autism Example: “Studies have shown that cannabis can lead to behavior improvements for children with autism”	Health claim
Neuropathy	Makes any health, medical, or therapeutic claims related to neuropathy Example: “Studies have shown that cannabinoids can reduce pain from neuropathy”	Health claim
IBS	Makes any health, medical, or therapeutic claims related to irritable bowel syndrome (IBS) Example: “Can cannabis help with IBS?” with link to article about how cannabis can improve IBS	Health claim
Crohns	Makes any health, medical, or therapeutic claims related to Crohn’s disease Example: “People with Crohn’s disease report improved symptoms after using cannabis”	Health claim
Migraine	Makes any health, medical, or therapeutic claims related to migraines	Health claim
Asthma	Makes any health, medical, or therapeutic claims related to asthma	Health claim
Alzheimers	Makes any health, medical, or therapeutic claims related to Alzheimer’s disease Excludes: General claims about memory or aging	Health claim
HIV/AIDS	Makes any health, medical, or therapeutic claims related to HIV/AIDS	Health claim
Dystonia	Makes any health, medical, or therapeutic claims related to dystonia	Health claim
Glaucoma	Makes any health, medical, or therapeutic claims related to glaucoma	Health claim
Lupus	Makes any health, medical, or therapeutic claims related to lupus	Health claim
Parkinsons	Makes any health, medical, or therapeutic claims related to Parkinson’s disease	Health claim
Seizures	Makes any health, medical, or therapeutic claims related to seizures	Health claim
TBI	Makes any health, medical, or therapeutic claims about traumatic brain injuries	Health claim
Tourette’s	Makes any health, medical, or therapeutic claims related to Tourette’s syndrome	Health claim
Colitis	Makes any health, medical, or therapeutic claims related to ulcerative colitis	Health claim
Arthritis	Makes any health, medical, or therapeutic claims related to arthritis	Health claim
Weight	Makes any health, medical, or therapeutic claims related to losing or maintaining weight. Examples include weight loss, maintaining a healthy weight, or treating/reducing obesity Excludes: weight gain from overeating, treating anorexia, or weight loss from an illness	Health claim
ALS	Makes any health, medical, or therapeutic claims related to amyotrophic lateral sclerosis (ALS)/Lou Gehrig’s disease	Health claim
Anorexia	Makes any health, medical, or therapeutic claims related to anorexia	
Fibro	Makes any health, medical, or therapeutic claims related to fibromyalgia	Health claim
Inflammation	Makes any health, medical or therapeutic claim related to reducing inflammation in general (not tied to other illness/disease/condition)	Health claim
Muscle	Makes any health, medical or therapeutic claim related to muscle relaxation and muscle soreness	Health claim
Exercise	Makes claim that using cannabis can enhance/improve exercise	Health claim
Memory	Makes general claim (not tied to specific illness) that cannabis use can improve memory or prevent memory loss	Health claim
Energy	Make claim that using cannabis will increase energy Excludes: alertness	Health claim
Mood	Makes general claim (not tied to specific illness) that cannabis use can improve/enhance mood Also includes: uplifting effects	Health claim
Productive	Makes claim that using cannabis will make you more productive	Health claim
Focus	Makes claim that using cannabis will improve focus or attention	Health claim
Relaxed	Makes claim that using cannabis will make you feel more relaxed, calm, soothed, makes you feel more carefree/worry-free, include “help make you more chill”	Health claim
Dehiscence	Makes any health, medical or therapeutic claim related to superior canal dehiscence syndrome	Health claim
Spinal	Makes any health, medical or therapeutic claim related to spinal cord injury	Health claim
Spino	Makes any health, medical or therapeutic claim related to spinocerebellar ataxia	Health claim
Arach	Makes any health, medical or therapeutic claim related to arachnoiditis	Health claim
Sjogren	Makes any health, medical or therapeutic claim related to Sjogren’s Syndrome	Health claim
Limb	Makes any health, medical or therapeutic claim related to residual limb pain	Health claim
Reflex	Makes any health, medical or therapeutic claim related to reflex sympathetic dystrophy	Health claim
Kidney	Makes any health, medical or therapeutic claim related to polycystic kidney disease	Health claim
Neurofib	Makes any health, medical or therapeutic claim related to neurofibromatosis	Health claim
Behcet	Makes any health, medical or therapeutic claim related to Neuro-Behcet’s disease	Health claim
Patella	Makes any health, medical or therapeutic claim related to nail-patella syndrome	Health claim
Myoclonus	Makes any health, medical or therapeutic claim related to myoclonus	Health claim
Gravis	Makes any health, medical or therapeutic claim related to myasthenia gravis	Health claim
MuscularD	Makes any health, medical or therapeutic claim related to muscular dystrophy	Health claim
Interstitial	Makes any health, medical or therapeutic claim related to interstitial cystitis	Health claim
Hydro	Makes any health, medical or therapeutic claim related to hydromyelia	Health claim
Hydrocephalus	Makes any health, medical or therapeutic claim related to hydrocephalus	Health claim
Hepc	Makes any health, medical or therapeutic claim related to hepatitis c virus	Health claim
FibroD	Makes any health, medical or therapeutic claim related to fibrous dysplasia	Health claim
EDS	Makes any health, medical or therapeutic claim related to Ehlers-Danlos syndrome	Health claim
Dyskinesia	Makes any health, medical or therapeutic claim related to dyskinesia	Health claim
CIDP	Makes any health, medical or therapeutic claim related to chronic inflammatory demyelinating polyneuropathy	Health claim
Syringomyelia	Makes any health, medical or therapeutic claim related to syringomyelia	Health claim
Arnold	Makes any health, medical or therapeutic claim related to Arnold Chiari malformation	Health claim
Causalgia	Makes any health, medical or therapeutic claim related to Causalgia	Health claim
Other_hc	Other health, medicinal, or therapeutic claim. Includes health claims related to animals. Exclude: vague references to medicine, healing, wellness	Health claim
Other_hc	Other health, medicinal, or therapeutic claim. Includes health claims related to animals. Exclude: vague references to medicine, healing, wellness	Health claim
Consumption	Depiction of someone consuming or in the process of consuming cannabis Also, includes: photos, drawings, cartoons, videos; animals or characters consuming cannabis Exclude: pre-rolls if not lit Examples: Video of someone using a bong, pictures of someone holding a vape pen as if they are in the process of smoking, cartoon person smoking, someone eating an edible cookie they baked in how-to video, someone holding a lit pre-roll as if they are smoking it	Other Violations
Leaf	Image of cannabis leaf or bud. For cannabis leaf, only include images that very clearly shown the iconic outline. For logos, do not code when just logo in profile pic, but do code when prominent in a post or picture Also includes: an image of a leaf or bud on a product Excludes: image of plant, variations in logos that look slightly like a leaf but not exactly	Other Violations
Overconsumption	Promotes overconsumption of cannabis products. Includes consuming edibles >10 mg in one sitting, promoting use every day, or references to being high all or most of time Examples: Shows or describes someone as or becoming “extremely high/stoned/baked”; joke about becoming “too high” to do X or “so high” that X happens	Other Violations
Bundling	Bundling different cannabis products for one price Excludes: bundling products for medical patients Example: vape pen and joint for $5	Other Violations
Give_away	Giving away cannabis products for free Excludes: buy one get one free sales, raffles that do not include cannabis products, giveaways for medical patients Examples: raffle, free samples, contest, lottery, sweepstakes	Other Violations
Games	Conducting games or competitions related to consumption Example: taking a hit of weed every time you lose in a game	Other Violations
Not_coded	Any post or tweet/retweet that is not coded anywhere else Examples: news article about the history of cannabis, interview with dispensary business owner, social equity loans (financing), cannabis industry profits, hours of operation, COVID guidelines	N/A

## Appendix D

**Table 6:** Comparison of non-compliance on Facebook and Twitter

	Facebook posts, %	Twitter posts, %	Difference, %
Sample size	*N* = 7793	*N* = 2668	
Any violation	32.8%	24.2%	8.6%*
Appealing to youth	10.7%	5.4%	5.3%*
Animal	5.3%	2.8%	2.5%*
Cartoon	4.9%	1.8%	3.2%*
Child	0.6%	0.6%	0.0%
Toy	0.5%	0.3%	0.2%
Likeness	2.0%	1.4%	0.6%*
Packaging	0.0%	0.0%	0.0%
Activity appealing to child	0.1%	0.0%	0.1%*
Health claim	11.5%	8.1%	3.4%*
Non-qualifying conditions	6.9%	4.2%	2.7%*
*Top 10 health claims*			
1. Anxiety	2.5%	1.1%	1.4%*
2. Pain	2.0%	2.3%	0.3%
3. Relaxing	2.1%	0.8%	1.2%*
4. Sleep	1.3%	2.3%	1.0%*
5. Chronic pain	1.5%	0.9%	0.6%*
6. Improve mood	1.2%	1.3%	0.0%
7. Inflammation	1.1%	1.4%	0.3%
8. Stress	1.1%	0.8%	0.3%
9. Cancer	0.9%	1.0%	0.0%
10. Depression	0.9%	0.8%	0.1%
Other violations	17.4%	14.5%	2.8%*
Depicts consumption	2.3%	0.8%	1.4%*
Image of leaf or bud	15.5%	12.2%	3.3%*
Promotes overconsumption	0.1%	0.6%	0.5%*
Bundling products for one price	0.2%	0.1%	0.1%
Giving away products	0.3%	1.1%	0.7%*
Conducting games/competitions	0.0%	0.0%	0.0%

Data were collected for the entire year of 2020. *Z*-tests were used to test for differences in the proportion of posts with violations from Facebook and Twitter accounts

^*^*p* < 0.05

## Abbreviations

THC: Tetrahydrocannabinol
FDA: Food and Drug Administration
PDF: Portable Document Format
CBD: Cannabidiol
MS: Multiple sclerosis
